# Guidelines for establishing an etching procedure for dislocation density measurements on multicrystalline silicon samples

**DOI:** 10.1016/j.mex.2018.09.013

**Published:** 2018-09-28

**Authors:** Krzysztof Adamczyk, Gaute Stokkan, Marisa Di Sabatino

**Affiliations:** a Department of Materials Science and Engineering, NTNU, NO-7491 Trondheim, Norway; b Sintef Materials and Chemistry, NO-7465 Trondheim, Norway

**Keywords:** Sopori etching for etch pit density measurements, Combined with PVScan and microscope image analysis, Selective etching, Sopori, Secco, Etch pit density, EPD, Dislocations, Silicon, Photovoltaic

## Abstract

With multicrystalline silicon becoming the main material used for photovoltaic applications and dislocations being one of the main material limitations to better solar cell efficiency, etch pit density measurements are gaining more importance. Traditionally, etch pit density measurements are based on selective etching of silicon samples. The majority of the etchants have been developed for monocrystalline samples with known orientation, while those developed for multicrystalline samples have been less investigated and might need some optimization. In this study, we use and compare the PVScan tool, which provides a quick way to assess dislocation density on selectively etched samples, and microscope image analysis. We show how the etching methods used for dislocation density measurements can affect the results, and we suggest how to optimize the Sopori etching procedure for multicrystalline silicon samples with high dislocation densities. We also show how the Sopori etchant can be used to substitute Secco while maintaining a high precision of dislocation density measurements, but without the toxic hexavalent chromium compounds.

## Graphical abstract

**Figure:**
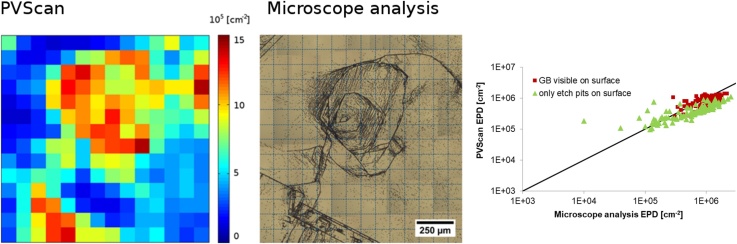

**Specifications Table**

**Table:** 

Subject area	*Materials Science*
More specific subject area	*Analysis of methods for dislocation density measurements in silicon material for photovoltaic applications*
Method name	*Sopori etching for etch pit density measurements, combined with PVScan and microscope image analysis*
Name and reference of original method	*Sopori etching* [1] *PVScan* [2] *ImageJ* [3]
Resource availability	• *Glassware and bench suitable for handling reagents with HF* • *Grinding machine, grinding disc with grain size 500 and 1200* • *Polishing machine, diamond paste with grain size 9, 3, 1 μm* • *Sopori etchant–* HF:CH_3_COOH:HNO_3_ with a ratio of 36:15:2 • *Secco etchant–HF:*015*mol K_2_Cr_2_O_7_with a ratio 2:1* • *PVScan 6000 measurement system* • *Metallographic microscope* • *GIMP – GNU Image Manipulation Program* • *ImageJ – Image Processing and Analysis in Java* • *Matlab – used for PVScan data imaging* • *MS Excel – used for result analysis and plotting*

## Background information

The Etch Pit Density (EPD) measured by the PVScan tool on an etched multicrystalline silicon wafer is typically in the range 10^4^ and 3 × 10^6^  cm^−2^. No lower and higher values are measured.

For smaller dislocation density areas, the reason for no detection might be that with the low density of etching pits a change in the reflected laser signal is too small to be measured. For larger dislocation density areas, like in dislocation clusters, correct dislocation density cannot be measured because the etch pits overlap.

The main motivation behind this work was to address these issues by modifying the etching time to obtain different size of dislocation etch pits and allow a more precise measurement with the fast, large-area PVScan technique. It was hypothesized that to measure intra-grain dislocations, which are in the range below 10^4^ cm^−2^, a possible way could be to etch the sample longer and obtain bigger etch pits. For dislocation clusters, decreasing the etching time should lead to smaller etch pits and less etch pit overlap.

In this work the influence of etching time on etch pit size and etch pit density measurements was evaluated. The tests presented in this work were performed with the Sopori etchant recipe. Several studies important for the PV industry have also been performed with the Secco etchant recipe containing a carcinogenic hexavalent chromium compound, potassium dichromate, K_2_Cr_2_O_7_ [4,5]. In addition to its toxicity, the Cr present in the Secco etchant can contaminate the sample and influence subsequent measurements of chemical composition analysis. Because of these factors, part of this work was also aimed at comparing the Secco and Sopori recipes for their use in etch pit density measurements to establish if the Sopori etchant could be used instead of Secco for precise measurements.

## PVScan and microscope image analysis for EPD measurements

All the measurements were performed on one 5 × 5 cm slab coming from a high performance multicrystalline silicon (HPMC-Si) ingot, seeded with Si feedstock from the fluidized bed reactor (FBR) process and solidified at NTNU/SINTEF lab [6]. To prepare the sample for etching, its surface was ground on sandpaper and polished using a diamond suspension down to 1 μm.

The standard recipe used at NTNU for selective etching consists of the steps presented below and performed in one etching session (ratios, if not stated otherwise, are given in units of volume):

1 RCA1 cleaning/10 min
2 Dip in deionized water
3 5% hydrofluoric acid - HF/3 min
4 Sopori/25 s
5 HF:HNO_3_(1:9)/5 s
6 Dip in deionized water
7 Flush with ethanol

The RCA1 cleaning mixture used in step 1 was developed in the Radio Corporation of America for cleaning of silicon wafers {Kern, 1990 #8} [7]. It consists of 5 parts of deionized water, 1 part of ammonia water (29% HNO_3_) and 1 part of aqueous H2O2 (30% H_2_O_2_). It is applied by dipping the silicon wafer in the mixture at a temperature of 80 °C with agitation. The remaining etchants and mixtures are applied by dipping the silicon wafer in the etchants at room temperature and without agitation, only with a very slow mixing introduced by moving the sample holder in the bath. The dipping time is given for each step. Dips in deionized water don’t require time control.

The Sopori etchant used in step 4 is a mixture of HF:CH_3_COOH:HNO_3_ with a ratio of 36:15:2. Silicon etching with such an etchant is a multi-reaction process in which a local concentration of reaction products could lead to increased etching locally. The etchant mixing by moving the sample holder in the bath is introduced to avoid such changes in the etching rate and allow for a more even distribution of substrates and products.

Evaluation of different etching time was performed by changing the time used for Sopori step in the standard NTNU recipe and leaving the remaining steps unchanged. In the first three etching sessions the sample was etched afterwards without repolishing, with Sopori etching steps lasting 5, 10 and 10 s – up to 25 s in total, as in the standard procedure. This was done to evaluate if it is possible to first obtain a measurement on a surface with small etch pits and subsequently etch the same sample further, to obtain measurements comparable with the standard procedure.

For the next sessions, the sample was repolished before each etching. Sessions with the Sopori step lasting 25, 75 and 150 s were performed to obtain standard and larger etch pits. Finally, the sample was repolished and etched for 5 s with the Sopori mixture diluted down to HF:CH_3_COOH:HNO_3_ ratio of 36:20:1 to obtain etch pits smaller than in the first three sessions.

The Secco etching procedure used in this work was as follows:

1 Dip in acetone
2 Dip in ethanol
3 Dip in deionized water
4 Secco/60 s

The Secco etchant is a mixture of one part of 0.15 M solution of K_2_Cr_2_O_7_ in H_2_O and two parts of 49% HF.

Etch pit density measurements with PVScan and microscope analysis was performed after each etching session.

**Fig. 1:**
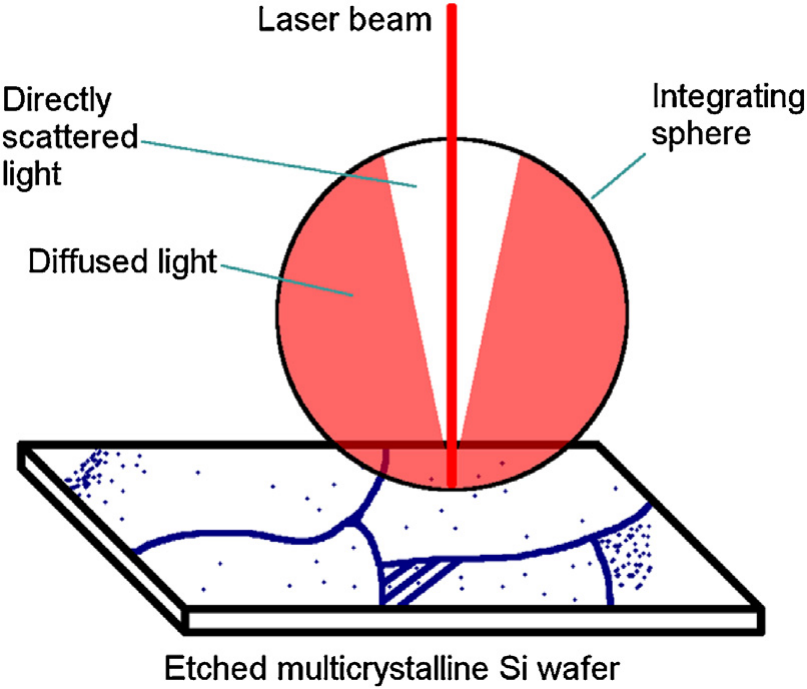
Schematic presenting the basic principle of PVScan operation [2].

PVScan 6000 is a surface scanning tool which allows for relatively fast evaluation of etch pit density by integrating the diffused light reflected by the etched surface. The basic principle of operation of this instrument is presented in Fig. 1. A laser beam is used to illuminate the surface. Surface free of any defects like dislocation clusters or grain boundaries will reflect the light directly and the signal coming from the diffused light detector is weak. When the laser illuminates defects, the signal is varied depending on the density of the defects. As mentioned earlier, this technique fails when the etch pits are not dense enough, or when their density is too high and leads to overlapping. Microscope image analysis allows to measure etch pit density more precisely, but requires more time and usually allows analysis of much smaller areas.

**Fig. 2:**
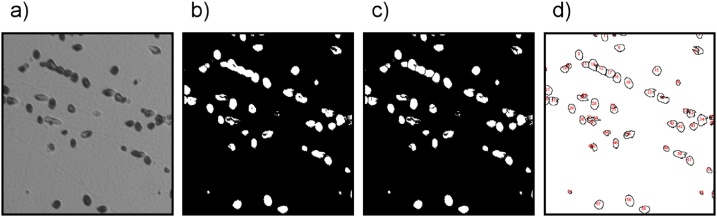
Etch pit density measurement by microscope image analysis with ImageJ software. The image shown here was slightly trimmed down from 100 × 100 μm for presentation. Its analysis resulted in a count of 58 etch pits, leading to a density of 5.8 × 10^5^ dislocations/cm^2^. Each frame shows an effect of a processing step. a) Microscope image is converted to 8-bit black and white image. b) Image is thresholded into 2 values – white for etch pit, black for background c) Watershed operation performed in order to divide overlapping etch pits d) Etch pits fulfilling the analysis conditions are counted by Particle Analysis tool.

Calculating the etch pit density by microscope image analysis can be explained with Fig. 2 showing the subsequent steps in the process. All the presented steps were performed in ImageJ software. The image was first converted from the color image format into a black and white image. This allowed for thresholding, which is a division of the pixels on image into two groups depending on their intensity values, higher and lower than the threshold, and assigning them to only two values, black and white. To compensate for etch pit overlap, the ‘watershed’ operation was performed on the image. This operation searches for cases of overlapping particles on the image, basing on their shape, and divides them accordingly. While it may lead to dividing single etch pits into several areas on the image if their shape is far from circular, the majority of the overlapping etch pits are separated. The etch pits fulfilling the analysis conditions are then counted by the software.

To compare both methods different areas were selected on the samples, one with large dislocation densities for analysis of shorter Sopori etching times, one with smaller dislocation densities for longer etching times. The selection was made on PVScan dislocation density maps which, while less precise, are easier and faster to obtain.

**Fig. 3:**
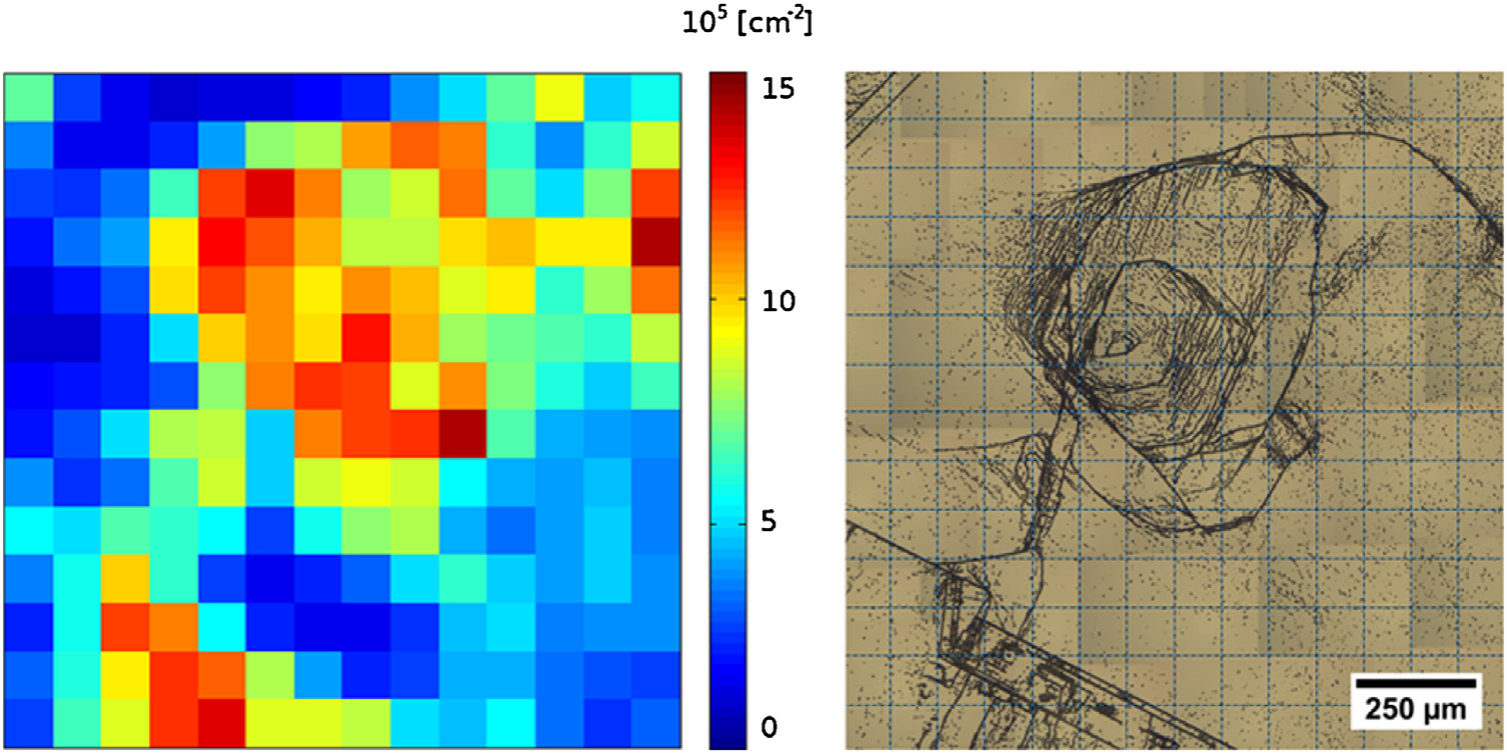
Images of same area of the silicon sample, obtained with different techniques: on the left is a PVScan etch pit density map and on the right is a microscope image, stitched and divided into areas corresponding to PVScan pixels. The orientation axis system on the PVScan map refers to pixel positions. Each such pixel corresponds to 100 × 100 μm square on the microscope image.

The dislocation density maps from PVScan are discrete, that is each value was obtained by stepping over the scanned area with a discrete step size. In the case of reported measurements this step size equaled 100 μm. Because of this the microscope images, covering a limited area, were first manually stitched in GIMP software into larger images covering areas which could be more easily compared with PVScan maps, and then divided into areas corresponding to PVScan pixels. This can be more easily understood when looking at Fig. 3. The resulting ‘pixel’ microscope images were automatically processed with ImageJ software, as described above. The etch pit density results were compared between each PVScan pixel and a corresponding ‘pixel’ from ImageJ microscope image analysis.

For the low dislocation density areas, the lowest density measured – that is if 1 etch pit was visible on 100 × 100 μm square – was equal to 10^4^ dislocations/cm^2^. Since lower values were expected in low dislocation density areas, they were divided into a grid of 200 x 200 μm instead of 100 × 100 μm. Such larger ‘pixels’ were compared with PVScan maps, which were recalculated to the same pixel size – one new pixel with a density value calculated as an average of four original PVScan pixels. Such binning allowed comparison with microscope image analysis of areas with etch pit densities down to 2.5 × 10^3^ dislocations/cm^2^. It needs to be noted that the PVScan laser beam has a diameter of about 800 μm, but reanalysing the microscope data so that each pixel result was a weighted average of itself and its surrounding pixels with weights accounting for the PVScan beam intensity did not lead to different conclusions than the ones without such recalculation, meaning that the intensity of the laser beam is not uniform and majority of PVScan signal comes from scattering mostly its high-intensity center part. For simplicity the microscope analysis results presented below are based on single pixels, without the weighted average recalculation.

Etch pit size was measured with ImageJ software on the microscope images obtained after etching.

**Fig. 4:**
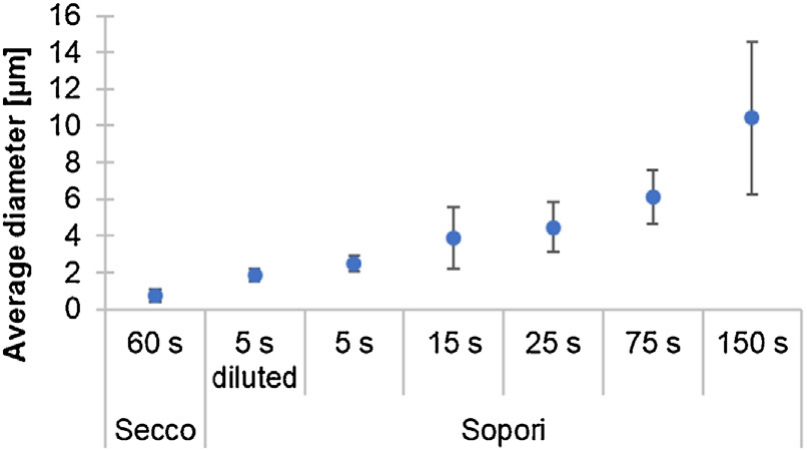
Etch pit size for each of the etching procedures tested in the experiment. 150 random etch pit diameters averaged for each etching time.

Etch pit size comparison is presented in Fig. 4. As expected for Sopori, the average etch pit diameter was largest for the longest etching time, and smallest for shortest time of etching in a dilute etchant. The etch pit size obtained according to the Secco etching procedure was smaller than the size obtained from a short etch in diluted Sopori.

**Fig. 5:**
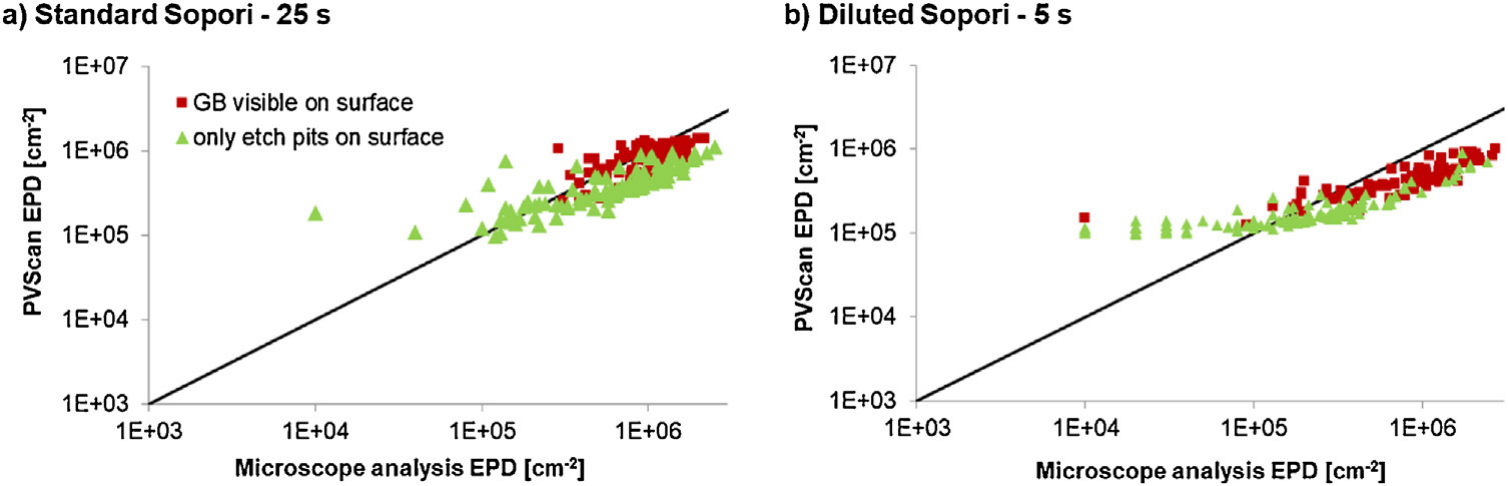
Charts presenting the comparison between dislocation density values measured by PVScan and by microscope image analysis. The different etching times were tested on the same area, repolished after each etching session. a) Standard NTNU Sopori etchant, used for 25 s as in standard procedure. b) Diluted Sopori etchant used only for 5 s, to obtain lower size of etch pits for analysis of high dislocation density areas.

**Fig. 6:**
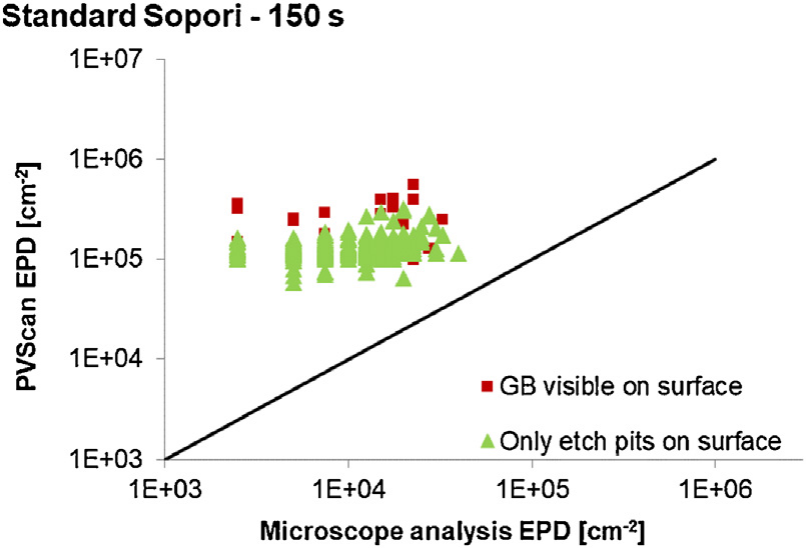
Comparison between dislocation density values measured by PVScan and by microscope image analysis on a sample etched with the standard Sopori etchant used for 150 s to obtain larger size of etch pits for analysis of low dislocation density areas.

The results showing a comparison between PVScan measurements and microscope image analysis with ImageJ are presented in Fig. 5, Fig. 6. The data for analysis of high dislocation density presented in Fig. 5 for both charts comes from the same area as presented in Fig. 4, while data for low dislocation density areas was obtained from a different sample area, free of larger dislocation clusters.

The black line dividing the chart area is a linear representation of the case where the results from PVScan are equal to the results from microscope image analysis. If the point is above this line, PVScan measured a value higher than obtained by microscope analysis on the same area. If the point is below the line, the PVScan value was lower. A distinction is made within the data between pixels covering an area with grain boundaries visible in addition to etch pits and pixels covering areas with etch pits, but without grain boundaries.

A general conclusion from the above comparison is that if a grain boundary is present in the scanned area, PVScan returns a higher dislocation density value than can be obtained with microscope analysis. The etched grain boundary also scatters the laser beam and more signal is measured by PVScan.

**Fig. 7:**
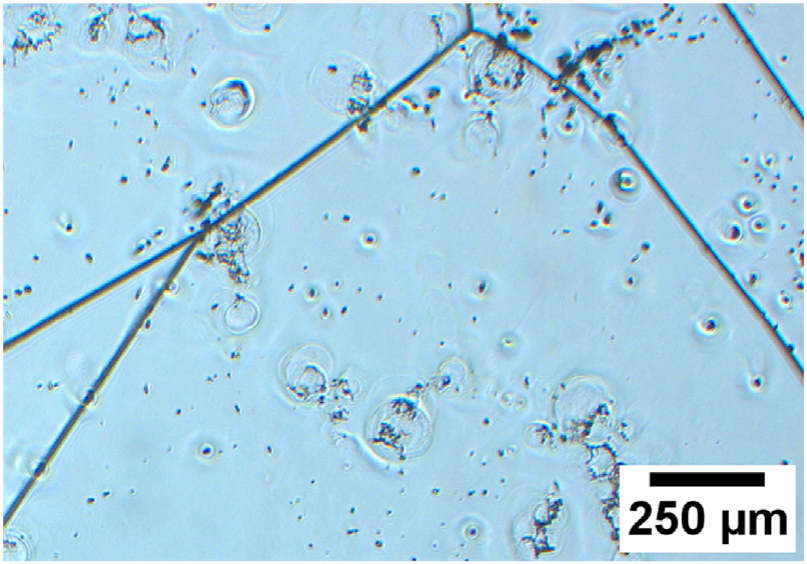
Image of sample surface after 150 s etching in standard Sopori etchant.

Another feature similar for all the datasets is that the PVScan showed a very low signal to noise ratio for dislocation densities below 10^5^ dislocations/cm^2^. This is a cutoff value for the data points on charts a) and b). Even for pixels where etch pits were visible with microscope in densities closer to 10^4^  cm^−2^, PVScan assigned values above 10^5^ cm^−2^. It was expected to have low signal to noise ratio coming from areas with low density of small etch pits, which is visible on the chart b) in Fig. 5, but increasing the etch pit size by longer etching did not result in better sensitivity of the PVScan system. The chart in Fig. 6 shows that there seems to be no correlation between dislocation density measured by PVScan and with microscope image analysis in the low density range. This might be related to the etching conditions, where the long etching time not only led to an increase in etch pit size, but also resulted in introducing significant artifacts on the sample surface. Some of these artifacts are visible in Fig. 7. As was seen in Fig. 4, the etch pits after 150 s of Sopori etching are not uniform, their size differs across a wide range of values. The reason for such artifacts can be that for such long etching times the mixing introduced by moving the sample in the bath is not efficient enough. Different techniques should be used to increase the mixing. The artifacts can affect the results from both techniques, with PVScan being probably more prone to error because of it. Each of these artifacts scatters light of the PVScan laser beam, resulting in increased signal for this technique. Majority of the artifacts is not counted as etch pits by the microscope image analysis algorithms due to their capability to distinguish defects by their size. Etching the sample with three subsequent steps without repolishing, with 5, 10 and 10 s steps, also led to having similar artifacts on the surface.

In the range of high dislocation densities, the data coming from a sample etched in a diluted etchant gave a smaller spread of values coming from PVScan. The PVScan settings are a linear calibration of the measured signal: ρ = C*S, where the dislocation density ρ is calculated from the signal S based on a calibration constant C [8]. The calibration constant can be adjusted for the diluted etchant showing a possibility of closer fit between PVScan and microscope image analysis in the range above 10^5^ cm^−2^. Modifying the calibration equation to contain a constant factor accounting for the noise in low dislocation density areas can be also considered. This would increase the precision of this fast measurement technique and make it more comparable with the more tedious, but also more precise microscope analysis.

**Fig. 8:**
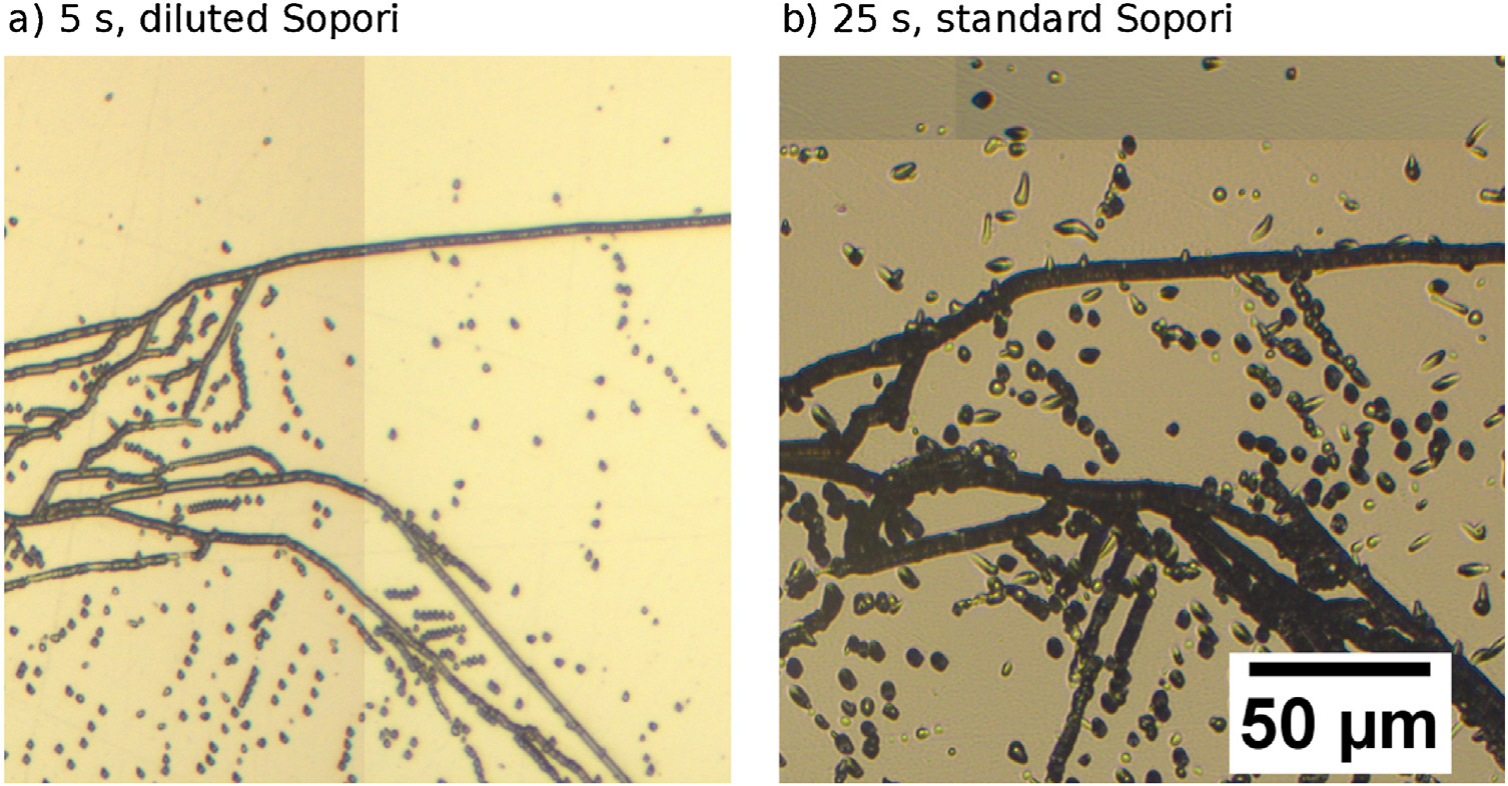
Microscope images of the same sample area, a) etched for 5 s in a diluted Sopori etchant and b) for 25 s in standard Sopori etchant. The sample was repolished between each etching session.

The comparison between measurements of high dislocation density areas showed an additional issue with precise measurement. As can be seen in Fig. 5, in the range below 10^5^ cm^−2^ there is a difference in what was found by microscope image analysis after 25 s in the standard etchant and after 5 s in a diluted etchant. As analysis of the etched surfaces reveals, the standard etch results in a variety of etch pits, ranging from circular to elliptic, and the elliptic etch pits seem more shallow. The difference in etch pit shape can be explained by differing angles between the surface and the dislocation core. The etch pits on the sample after the shorter, diluted etch are much more uniform, as if only the dislocations at a certain preferential angle to the surface were etched. The small etch pit size with fewer overlapping etch pits allowed for a more precise quantification of high dislocation density areas, but the fact that part of the dislocations aligned in less preferential orientations didn’t etch needs to be considered when applying this technique. A comparison of the surface after the two discussed etching steps is shown in Fig. 8.

As the above results show, dividing the etching procedure into short etching for high dislocation density analysis and subsequent long etching for low dislocation density analysis does not yield good results, especially in the low density range after long etching. Combining long etching with short etching without repolishing samples between such sessions may introduce etching artifacts and make further measurements unprecise. Shorter etching times can be applicable if only high dislocation density areas need to be characterized with more precision. Proper optimization of the etching time can allow precise analysis on the PVScan instrument.

## Comparison between Sopori and Secco etching

**Fig. 9:**
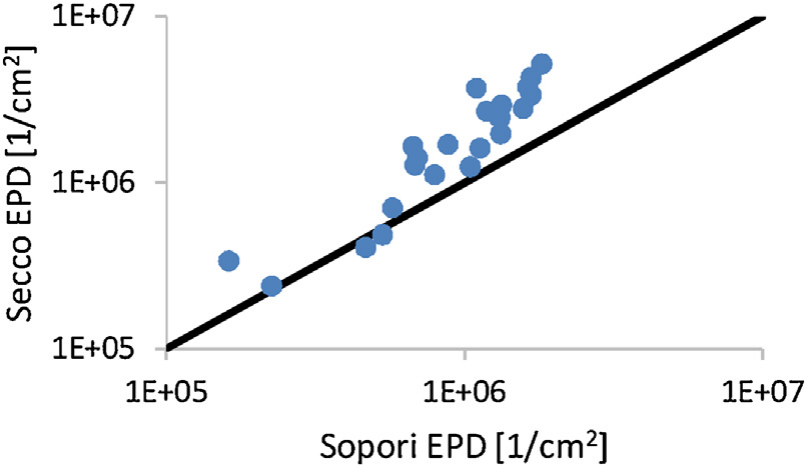
Effect of etching procedure on EPD measurements by microscope image analysis. 60 s Secco and 5 s standard Sopori are compared.

For a comparison between the Secco etchant and Sopori etchant, the etch pit density was measured in the same areas after etching for 5 s in standard Sopori and then after repolishing and etching in Secco for 60 s. In the original paper on Secco it was suggested to etch monocrystalline silicon samples for 5 min with ultrasonic agitation. 60 s without agitation is enough to selectively etch multicrystalline silicon samples, thus 60 s was used for this comparison. The results of the microscope image analysis of the surface obtained after each of these procedures are shown in 9. Microscope image analysis was chosen as the technique for this comparison, because due to the etch pit size the PVScan signal was very weak, resulting in lower dislocation densities. Fig. 9 indicates that even 5 s is too much for the Sopori etchant to obtain precise dislocation density measurements in the high density range. The 60 s Secco etch gives an etch pit size of about 0.5–1 μm, while the 5 s Sopori etch results in 2 μm etch pits. The main cause for the difference in measurement between the two etching procedures is the overlap of etch pits in the range above 10^5^ cm^−2^ for Sopori.

The conclusion from this comparison is that the Secco etchant can be replaced with Sopori etching, but the Sopori etching procedure needs to be further optimized for measurements of high dislocation density areas. Etching times with the diluted Sopori etchant below 5 s are suggested in such case. Replacing the Secco etchant with Sopori gives significant benefits since one can avoid toxic and carcinogenic Cr compounds.
